# cPLA2*α* mediates TGF-*β*-induced epithelial–mesenchymal transition in breast cancer through PI3k/Akt signaling

**DOI:** 10.1038/cddis.2017.152

**Published:** 2017-04-06

**Authors:** Lu Chen, Hui Fu, Yi Luo, Liwei Chen, Runfen Cheng, Ning Zhang, Hua Guo

**Affiliations:** 1 Department of Tumor Cell Biology, Tianjin Medical University Cancer Institute and Hospital, Tianjin 300060, China; 2 The Key Laboratory of Tianjin Cancer Prevention and Treatment, National Clinical Research Center for Cancer, Tianjin 300060, China; 3 Department of Pathology, Tianjin Medical University Cancer Institute and Hospital, Tianjin 300060, China

## Abstract

A high incidence of tumor recurrence and metastasis has been reported in breast cancer patients; nevertheless, the underlying molecular mechanisms are largely unknown. Epithelial–mesenchymal transition (EMT), which is induced by transforming growth factor-*β* (TGF-*β*), has been implicated in tumorigenesis and breast cancer metastasis. EMT events are now directly associated with tumor metastasis, and this progress is dependent on the inflammatory microenvironment. Cytosolic phospholipase A2*α* (cPLA2*α*) has been shown to participate in a series of biological processes including inflammation and cancer development. However, the role and molecular mechanism of cPLA2*α* in breast cancer EMT and metastasis remain enigmatic. In this study, we found that cPLA2*α* was commonly overexpressed in most human breast cancer tissues and significantly correlated with a poor prognosis for human breast cancer. Functional studies demonstrated that cPLA2*α* overexpression was significantly associated with elevated migration and invasion in MDA-MB-231 and T47D cells. Conversely, reduced cPLA2*α* expression strongly attenuated metastasis and the EMT program of MDA-MB-231 cells. Further study found that knockdown of cPLA2*α* in MDA-MB-231 cells inhibited TGF-*β*-induced EMT through the PI3K/Akt signaling pathway. Animal experiments revealed that cPLA2*α* downregulation in MDA-MB-231 cells markedly restrained tumorigenesis and metastasis *in vivo*. This study indicates the potential role of cPLA2*α* in breast cancer metastasis and indicates that this molecule is a promising therapeutic target for breast cancer.

Breast cancer is one of the most commonly diagnosed cancers in women worldwide.^1^ It can be divided into four intrinsic molecular subtypes including luminal A, luminal B, human epidermal growth factor receptor-2 (HER2)-enriched and triple negative by immunohistochemical analyses of estrogen receptor, progesterone receptor and HER2.^2^ Recurrence and metastasis are the main causes of fatality associated with breast cancer.^3^ Therefore, there is a desperate need to reveal the mechanisms underlying breast cancer metastasis.

The tumor microenvironment is a complex system of both cellular and subcellular components with reciprocal signaling that contributes critically to the carcinogenic process.^4^ The inflammatory microenvironment is a key component of tumors.^5^ In the inflammatory environment, activated immune cells are capable of producing factors such as transforming growth factor-*β* (TGF-*β*), tumor necrosis factor-*α* and interleukin-6 that induce epithelial–mesenchymal transition (EMT).^6, 7, 8^ These characteristics suggest that inflammation has an important role in tumor EMT.^9^

Phospholipase A2 represents a superfamily of enzymes that can catalyze the hydrolysis of the fatty acyl ester bond at the sn-2 position of phospholipids to produce free fatty acids and lysophospholipids.^10^ These lipids are also substrates for intracellular biochemical pathways that generate lipid mediators, which may act as crucial mediators between chronic inflammation and cancer.^11, 12, 13, 14, 15^ Of the known mammalian PLA2 enzymes, cytosolic PLA2*α* (cPLA2*α*) is an 85-kDa protein with a high selectivity for liberating arachidonic acid (AA), which can be metabolized by cyclooxygenase (COX) enzymes and subsequently converted to a panel of eicosanoid production.^16^ Many studies have shown that COX2 is associated with tumor invasion and metastasis via EMT in many tumors such as bladder cancer,^17^ hepatocellular carcinoma (HCC),^18^ cutaneous squamous cancer^19^ and breast cancer.^20^ cPLA2*α* has a role in many biological processes, including inflammation,^10^ cell growth and cancer development.^21, 22, 23, 24^ Many studies have shown that cPLA2*α* is synchronously overexpressed and participates in tumorigenesis through producing sufficient substrates for the metabolic cascade of COX2/PGE2 and other pathways.^25, 26, 27, 28^ However, the functions of cPLA2*α* in breast cancer migration and invasion remain to be elucidated.

Previous reports have indicated that TGF-*β* could regulate the growth of hepatocytes through phosphorylation of cPLA2*α.*^29^ Furthermore, more evidence has shown that TGF-*β* induced phosphorylation of PI3K/Akt/mTOR pathway to promote EMT transition.^29^ In this study, we demonstrated that cPLA2*α* expression positively correlated with lymph node metastasis, tumor relapse, histological grade and poor prognosis. Furthermore, we demonstrated that cPLA2*α* induced breast cancer cells to undergo EMT via the PI3K/Akt signaling pathway. Our results unraveled a novel function of cPLA2*α* as a mediator of EMT and migration in breast cancer cells, implicating it as a potential target for the prevention of tumor metastasis.

## Results

### cPLA2*α* was overexpressed in breast cancer tissues and invasive breast cancer cell lines

**Figure 1:**
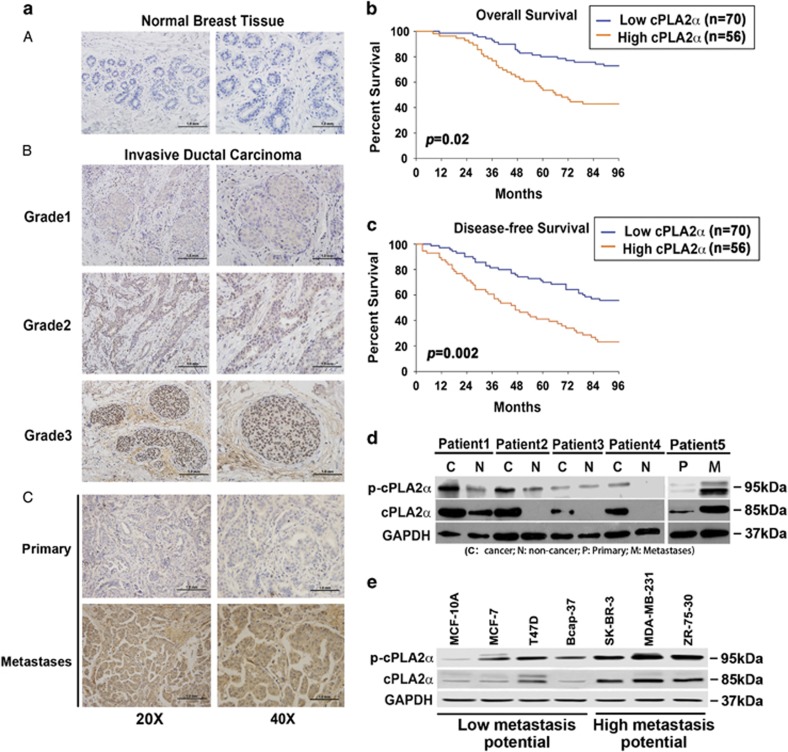
cPLA2*α* were overexpressed in breast cancer tissues and invasive breast cancer cell lines. (**a**) A – Immunohistochemistry of cPLA2*α* in mammary gland flocculus hyperplasia tissues; B – immunohistochemistry of cPLA2*α* in breast cancer tissues with different histological grades; c – immunohistochemistry of cPLA2*α* in primary breast cancer tissues and distant metastasis tumor tissues. (**b**) Kaplan–Meier overall survival analysis of 126 patients with high or low cPLA2*α* expression. (**c**) Kaplan–Meier disease-free survival analysis of 126 patients with high or low cPLA2*α* expression. (**d**) Western blot of cPLA2*α* and p-cPLA2*α* in four pairs of fresh-frozen tissues containing immunohistochemical cancerous tissues and non-cancerous tissues and one pair of primary cancerous tissues and metastasis tissues from breast cancer patient (C: cancer; N: non-cancer; P: primary; M: metastases). Subtypes of different breast cancer patients: Patient 1 (HER2-enriched), Patient 2 (triple negative), Patient 3 (triple negative), Patient 4 (luminal A) and Patient 5 (luminal A). (**e**) The protein level of cPLA2*α* and p-cPLA2*α* in different breast cancer cell lines and normal mammary epithelial cell line MCF-10A. Subtypes of different breast cancer cell lines: MCF-7 (luminal A); T47D (luminal A); Bcap-37 (luminal A); SK-BR-3 (HER2-enriched); MDA-MB-231 (triple negative); ZR-75-30 (triple negative)). Scale bar, 1.0 mm

To evaluate the role of cPLA2*α* in human breast cancer, we determined cPLA2*α* expression using immunohistochemistry in 126 patients with different histological grades of invasive ductal breast cancer and 20 patients with mammary gland flocculus hyperplasia, who underwent surgery at the Tianjin Medical University Cancer Hospital. None of the patients had been treated with other methods, such as chemotherapy or radiotherapy, before the operation. The results showed that cPLA2*α* expression was markedly overexpressed in human breast cancer tissues compared with the mammary gland flocculus hyperplasia tissues (Figures 1aA and B). The increased cPLA2*α* expression was significantly associated with the histological grades of breast cancer (*P*=0.001; Figure 1aB). Moreover, we further detected cPLA2*α* expression using immunohistochemistry in six patients with primary breast cancer tissues and distant metastases tissues, which included breast cancer thoracic vertebra metastases (1 case), breast cancer lung metastases (2 cases), breast cancer liver metastases (1 case), breast cancer jejunal metastases (1 case) and breast cancer frontal lobe metastases (1 case). The results showed that cPLA2*α* expression was markedly overexpressed in metastases tissues compared with the primary breast cancer tissues in the same patients (Figure 1aC).

Clinicopathological analysis showed that cPLA2*α* expression was linked with tumor size, lymph node metastasis, tumor relapse and histological grade (Table 1). Out of 55 cases of lymphonodus metastasis-positive patients, 33 (60%) were associated with positive cPLA2*α* expression. Simultaneously, out of the 71 cases of lymphonodus metastasis-negative patients, 23 (32.39%) were cPLA2*α* positive. At the same time, among the 83 cases of patients with a tumor of a diameter >2 cm, 47 (56.63%) were cPLA2*α* positive, whereas, among the 43 cases of patients with a tumor of a diameter ⩽2 cm, 9 (20.93%) were cPLA2*α* positive. Differences of lymphonodus metastasis (*P*=0.002) and tumor size (*P*<0.001) between the two groups were significant (Table 1).

Clinical survival analysis indicated that the patients with low cPLA2*α* expression level had a significantly better outcome either in overall survival (*P*=0.02) or in disease-free survival (*P*=0.002) (Figures 1b and c). Then, we extracted the protein from fresh-frozen tissues from four breast cancer patients who underwent surgery in our hospital. Western blot showed that both cPLA2*α* and p-cPLA2*α* expression levels were significantly higher in cancerous tissue than in the adjacent non-cancerous tissue from the same patient (Figure 1d). Additionally, the expression level of cPLA2*α* and p-cPLA2*α* in metastasis tumor tissues was markedly higher than in primary tumor tissues from the same patient (Figure 1d). This finding was similar to the result of the immunohistochemical assay. Furthermore, we determined cPLA2*α* and p-cPLA2*α* expression in different breast cancer cell lines and found that the increased cPLA2*α* and p-cPLA2*α* expression was associated with the metastatic potential of each cell line (Figure 1e).

### Inhibition of cPLA2*α* impaired breast cancer cell migration

To investigate the role of cPLA2*α* in breast cancer behavior, we used a cPLA2*α*-specific inhibitor (Merck-Calbiochem, Darmstadt, Germany; 525143-500UGCN) with MDA-MB-231 cells. At the same time, a phospholipase C inhibitor was used as a control. The results demonstrated that the specific inhibition of cPLA2*α* significantly impaired breast cancer cell chemotaxis and migration (Supplementary Figures 1A and B). Then, we verified the influence of cPLA2*α* inhibitor on another breast cancer cell line, T47D, which showed a similar result as that observed in the MDA-MB-231 cells (Supplementary Figure 1C). To investigate the functions of cPLA2*α* in breast cancer, we knocked down cPLA2*α* expression using siRNA transient transfection in MDA-MB-231 cells, and the specificity of cPLA2*α* knockdown was examined by western blot and qRT-PCR (Supplementary Figure 1D). In the subsequent chemotaxis assay, we found that all three siRNAs inhibited MDA-MB-231 cell chemotaxis (Supplementary Figure 1E). Similar studies were performed in T47D cells (Supplementary Figure 1F), which demonstrated the same effect.

These results indicated that silencing of cPLA2*α* by siRNA transient transfection and a cPLA2*α*-specific inhibitor significantly depressed breast cancer cell migration and chemotaxis.

### cPLA2*α* overexpression enhanced breast cancer cell migration and invasion *in vitro*

**Figure 2:**
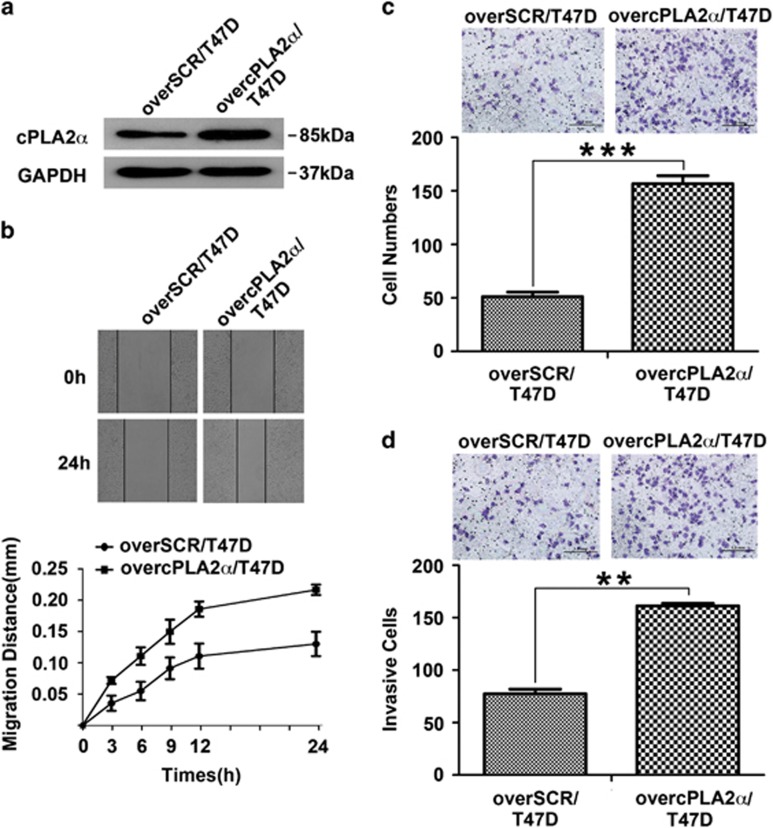
cPLA2*α* overexpression enhanced breast cancer cells migration and invasion *in vitro*. (**a**) Assessment of the transfected efficiency of cPLA2*α* protein expression after retroviral infection in T47D cells. (**b**) Scratch assay comparing the migration of SCR/T47D (left) and overcPLA2*α*/T47D (right) cells. (**c**) Comparison of chemotaxis potential of SCR/T47D (left) and overcPLA2*α*/T47D (right) cells under the application of high concentration of FBS (20%) with constant stimulation for 6 h. (**d**) Comparison of the invasion potential of SCR/T47D (left) and overcPLA2*α*/T47D (right) cells after incubation with EGF (10 ng/ml) at 37 °C in 5% CO_2_ for 36 h by counting the number of the cells that invaded through Matrigel-coated transwell inserts. **P<0.01, ***P<0.001. Scale bar, 1.0 mm. All experiments were repeated at least three times

To confirm the effect of cPLA2*α* on regulating migration and invasion processes, T47D cells were selected to establish stable cPLA2*α*-overexpressing cells using specific plasmid lentiviral infection. The efficiency of cPLA2*α* overexpression was verified by western blot (Figure 2a). A wound-healing assay showed that overexpressed cPLA2*α* in T47D cells led to a faster migration rate than that in the control group (Figure 2b). Chemotaxis and transwell assays showed that overexpressed cPLA2*α* in T47D cells markedly increased the number of migrated and invaded cells (Figures 2c and d).

In summary, our results indicated that cPLA2*α* could regulate human breast cancer cell migration and invasion.

### cPLA2*α* regulated MDA-MB-231 cell migration, chemotaxis and invasion capacities *in vitro*

**Figure 3:**
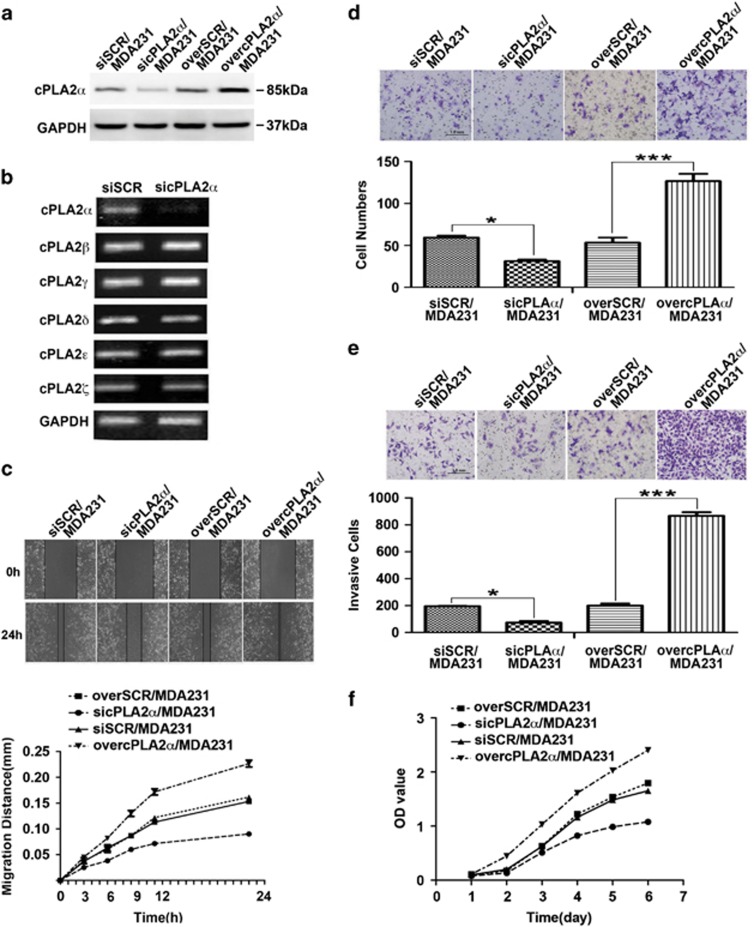
cPLA2*α* regulated MDA-MB-231 cell migration and invasion capacities *in vitro*. (**a**) Assessment of the transfected efficiency of cPLA2*α* protein expression after retroviral infection in MDA-MB-231 cells. (**b**) Repression specificity of cPLA2*α* mRNA expression in MDA-MB-231 cells by qRT-PCR. (**c**) Scratch assay comparing the migration of SCR/MDA-MB-231, sicPLA2*α* /MDA-MB-231 and overcPLA2*α*/MDA-MB-231 cells. (**d**) Comparison of chemotaxis potential of SCR/MDA-MB-231, sicPLA2*α* MDA-MB-231 and overcPLA2*α*/MDA-MB-231 cells under the application of high concentration of FBS (20%) with constant stimulation for 4 h. (**e**) Comparison of the invasion potential of SCR/MDA-MB-231, sicPLA2*α*/MDA-MB-231 and overcPLA2*α*/MDA-MB-231 cells after incubation with EGF (10 ng/ml) at 37 °C in 5% CO_2_ for 24 h by counting the number of the cells that invaded through Matrigel-coated transwell inserts. (**f**) Comparison of the proliferation of SCR/MDA-MB-231, sicPLA2*α*/MDA-MB-231 and overcPLA2*α*/MDA-MB-231 cells by Cell Counting Kit-8 (CCK8) assay. **P<0.01 and ***P<0.001. Scale bar, 1.0 mm. All experiments were repeated at least three times

To further investigate the role of cPLA2*α* in cancer cell migration, chemotaxis and invasion, we stably depressed and overexpressed cPLA2*α* in MDA-MB-231 cells using specific plasmid lentiviral infection. The transfected efficiencies were confirmed by western blot (Figure 3a). Considering that the cytosolic phospholipase A2 superfamily has six different subtypes, cPLA2*α*, cPLA2*β*, cPLA2*θ*, cPLA2*δ*, cPLA2*ɛ* and cPLA2*γ*, we further confirmed transfection using real-time PCR (Figure 3b). Wound-healing assays showed that overexpressed or silenced cPLA2*α* in MDA-MB-231 cells enhanced or inhibited the migratory activity of the MDA-MB-231 cells, respectively (Figure 3c). The analysis of cells' chemotactic movements and invasion ability using chemotaxis and Matrigel invasion assays showed similar results. cPLA2*α* depletion in MDA-MB-231 cells evidently reduced cell migration and invasion. In contrast, these capacities were markedly enhanced when cPLA2*α* was overexpressed in MDA-MB-231 cells (Figures 3d and e). The result of cell proliferation assay showed that the deletion of cPLA2*α* in breast cancer cells slowed down tumor cell proliferation, whereas upregulated cPLA2*α* in breast cancer cells promoted tumor cell proliferation (Figure 3f). However, the rates of apoptosis determined by flow cytometry analysis of Annexin V-FITC/PI showed no difference between the groups (Supplementary Figures 2A and B).

### cPLA2*α* promoted EMT in breast cancer cells

**Figure 4:**
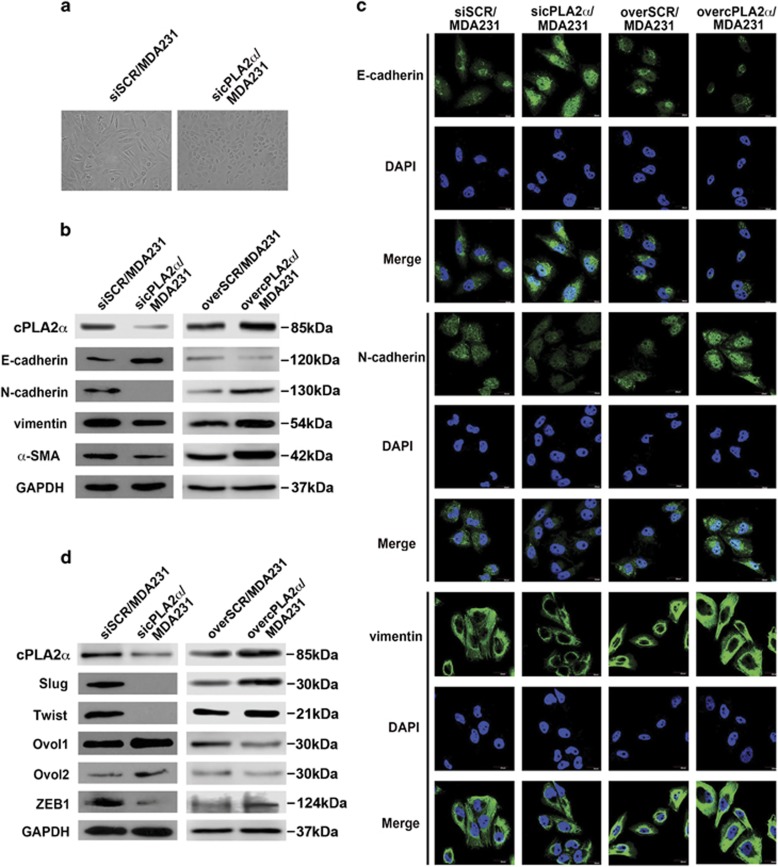
The depression of cPLA2*α* inhibited TGF-*β*-induced EMT and promoted MDA-MB-231 cells occurring MET. (**a**) Morphologic change of the MDA-MB-231 cells when cPLA2*α* was endogenously knocked down. (**b**) Western blot of the expression of EMT-associated markers (epithelial marker E-cadherin, mesenchymal markers N-cadherin, vimentin and *α*-SMA) in MDA-MB-231 cells when cPLA2*α* was knocked down or overexpressed, respectively. (**c**) Immunofluorescence staining for EMT-associated markers (epithelial marker E-cadherin, mesenchymal markers N-cadherin, vimentin) in SCR/MDA-MB-231, sicPLA2*α*/MDA-MB-231 and ovecPLA2*α*/MDA-MB-231 cells, respectively. Scale bar, 20 *μ*m. All experiments were repeated at least three times. (**d**) Compared EMT-associated transcription factors (Slug, Twist and ZEB1) and MET-associated transcription factors (OVOL1 and OVOL2) expression between siSCR/MDA-MB-231 and sicPLA2*α*/MDA-MB-231 cells (left); overSCR/MDA-MB-231 and overcPLA2*α*/MDA-MB-231 cells (right) by western blot assay, respectively. Scale bar, 20 *μ*m

After cPLA2*α* was stably knocked down in MDA-MB-231 cells, we observed that sicPLA2*α*/MDA-MB-231 cells showed morphologic changes from elongated fibroblastoid phenotype to polarized epithelial phenotype with increased cell–cell close connections compared with MDA-MB-231 cells (Figure 4a). Based on these phenomena, we speculated that cPLA2*α* may have a role in breast cancer cell EMT, which has been demonstrated to have an important role in cancer cell migration and invasion.^30^ Then, we verified these morphological changes using western blot and immunofluorescence through a series of markers related to EMT. The data showed that silenced cPLA2*α* in MDA-MB-231 cells increased the expression of epithelial marker E-cadherin while decreasing the expression of the mesenchymal markers, such as N-cadherin, vimentin and *α*-SMA (Figure 4b). We further confirm the role of cPLA2*α* in regulating the EMT or the mesenchymal–epithelial transition (MET) processes in overcPLA2*α*/MDA-MB-231 cells. We found that the epithelial marker E-cadherin was remarkably downregulated, whereas the mesenchymal markers were upregulated (Figure 4b). Similar results were obtained using an immunofluorescence assay. Overexpressed cPLA2*α* in MDA-MB-231 cells promoted the EMT process, whereas the deficiency of cPLA2*α* promoted the MET process in breast cancer cells (Figure 4c).

Then, we examined EMT-associated transcription factors in MDA-MB-231 cells. We found that depletion of endogenous cPLA2*α* in MDA-MB-231 cells suppressed the expression of Slug, Twist and ZEB1, which are known to act as EMT promoters. Depletion of cPLA2*α* also caused the overexpression of Ovol1 and Ovol2 in MDA-MB-231 cells, which are known to be the MET-driving promoters (Figure 4d). In contrast, overexpressed cPLA2*α* in MDA-MB-231 cells promoted Slug, Twist and ZEB1 expression and silenced Ovol1 and Ovol2 expression compared with the control group (Figure 4d).

Considering our findings as mentioned above, we came to the conclusion that cPLA2*α*-regulated breast cancer cell EMT and MET transition processes through a group of EMT- and MET-associated transcription factors.

### Activated cPLA2*α*-mediated TGF-*β*-induced EMT through PI3K/Akt/GSK-3*β*/*β*-catenin pathway

Next, we sought to determine the signaling mechanisms involved in the cPLA2*α*-mediated EMT process. TGF-*β* has been considered as an effective inducer and a maintenance factor of EMT, and it has been shown that TGF-*β* is responsible for cancer cell metastasis.^31, 32^ Certain signaling pathways, such as MAPK/ERK, Wnt, PI3K/Akt and Rho GTPase, have been shown to have an important role in the EMT process.^33, 34, 35, 36^ Actually, the activation of focal adhesion kinase (FAK) is one of the most important central steps for regulating downstream signaling pathways that control cell spreading, chemotaxis and invasion.^37^ Recent data have suggested that FAK activation could further activate PI3K cascades.^38^ Therefore, we hypothesized that TGF-*β* might mediate EMT in breast cancer cells through cPLA2*α* by activating the FAK/PI3K/Akt/GSK-3*β* pathway.

**Figure 5:**
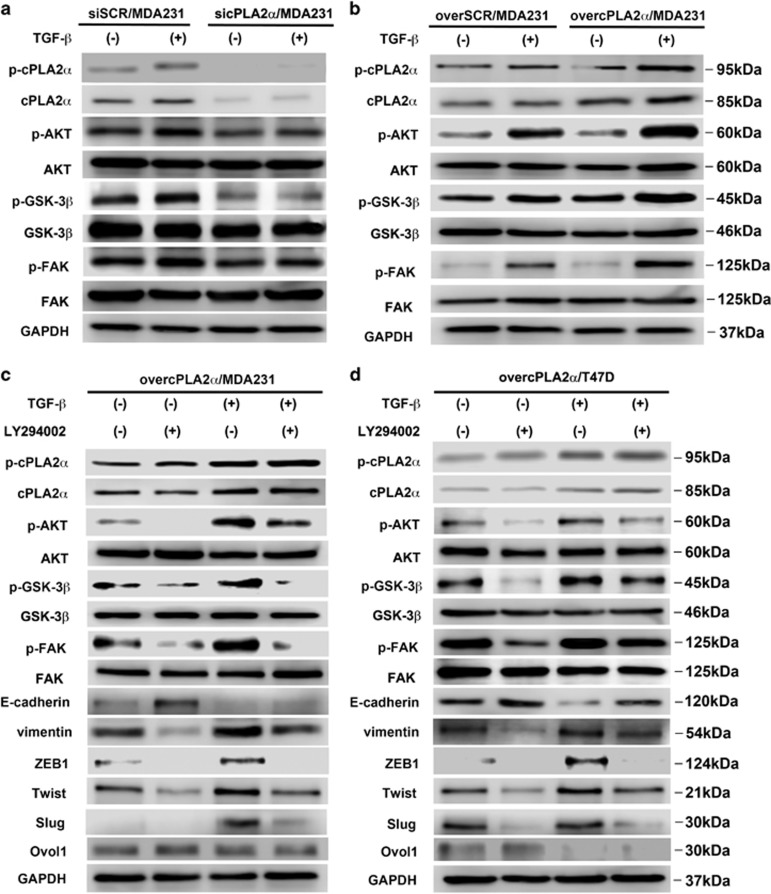
Activation of cPLA2*α* mediated TGF-*β*-induced EMT through the PI3K/Akt/GSK-3*β* pathway. Western blot of phosphorylated cPLA2*α*, FAK, Akt, GSK-3*β* and total cPLA2*α*, FAK, Akt, GSK-3*β* in (**a**) siSCR/MDA-MB-231 and sicPLA2*α*/MDA-MB-231 cells when cultured without or with TGF-*β* (5 ng/ml, 72 h); (**b**) overSCR/MDA-MB-231 and overcPLA2*α*/MDA-MB-231 cells when cultured without or with TGF-*β* (5 ng/ml, 72 h); western blot of phosphorylated cPLA2*α*, FAK, Akt, GSK-3*β*; total cPLA2*α*, FAK, Akt, GSK-3*β* and EMT-associated markers and transcription factors in (**c**) overcPLA2*α*/MDA-MB-231 cells when cultured without or with TGF-*β* (5 ng/ml, 72 h) and LY294002; (**d**) overcPLA2*α*/T47D cells when cultured without or with TGF-*β* (5 ng ng/ml, 72 h) and LY294002. All experiments were repeated at least three times

The western blot results showed that downregulation of cPLA2*α* in MDA-MB-231 cells remarkably decreased the phosphorylation of FAK, Akt and GSK-3*β*, whereas it had no influence on total FAK, Akt and GSK-3*β* expression (Figure 5a). cPLA2*α* suppression strongly affected the TGF-*β*-induced phosphorylation of FAK, Akt and GSK-3*β* in MDA-MB-231 cells (Figure 5a). In contrast, the stable overexpression of cPLA2*α* in MDA-MB-231 cells increased the phosphorylation levels of FAK, Akt and GSK-3*β*, and the influence of TGF-*β* in inducing phosphorylation was noticeable (Figure 5b). The results also showed that both total cPLA2*α* and phosphor-cPLA2*α* expression levels were elevated by the stimulation of TGF-*β* (Figures 5a and b).

When compared with Akt and FAK, we found both basal and TGF-*β*-induced phosphorylation expression of GSK-3*β* seem to require cPLA2*α* (Figure 5a). As GSK-3*β* is a critical regulator of *β*-catenin stability, nuclear translocation and EMT, we further detected *β*-catenin nuclear levels in response to cPLA2*α*. The breast cancer cells were incubated with TGF-*β* for 2 h. The result showed that TGF-*β* was capable of inducing the nuclear translocation of *β*-catenin and the effect was more significant, especially in overcPLA2*α*/MDA231 cells when compared with SCR/MDA231 cells and sicPLA2*α*/MDA231 cells (Supplementary Figures 3A and B). Moreover, without TGF-*β* stimulation, overexpressed cPLA2*α* in MDA231 cells could also improve *β*-catenin nuclear translocation (Supplementary Figures 3A and B). Furthermore, downregulation of *β*-catenin in overcPLA2*α*/MDA231 cells markedly inhibited cells' chemotactic movements (Supplementary Figures 3C and D). We noticed some differences of proliferation between two groups but they did not achieve statistical significance (Supplementary Figure 3E).

To further confirm that cPLA2*α* regulated the EMT process through the PI3K pathway, we cultured overcPLA2*α*/MDA-MB-231 and overcPLA2*α*/T47D cells with an exogenous specific inhibitor of PI3K (LY294402). The results showed that LY294002 markedly inhibited the upregulated phosphorylation of FAK, Akt and GSK-3*β* compared with the control group in overcPLA2*α*/MDA-MB-231 cells and the function of TGF-*β* in inducing phosphorylation was balanced (Figure 5c). At the same time, the mesenchymal marker vimentin as well as the EMT promoters ZEB1, Twist and Slug were upregulated, whereas the epithelial marker E-cadherin as well as the MET-driving promoter Ovol1 were downregulated in overcPLA2*α*/MDA-MB-231 cells (Figure 5c). A similar phenomenon was observed in overcPLA2*α*/T47D cells (Figure 5d).

Taken together, the data suggested that TGF-*β* contributed to the MDA-MB-231 cell EMT process through cPLA2*α* by activating the FAK/PI3K/Akt/GSK-3*β***/***β*-catenin pathway.

### Knockdown of cPLA2*α* in MDA-MB-231 cells suppressed tumorigenesis and inhibited lung metastasis *in vivo*

**Figure 6:**
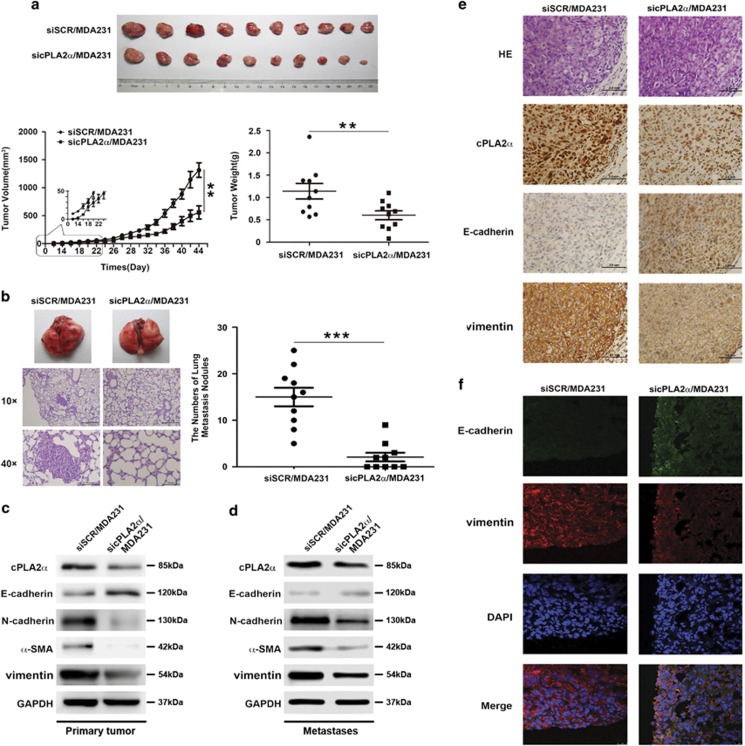
Knockdown of cPLA2*α* in MDA-MB-231 cells suppressed tumorigenesis and inhibited metastasis *in vivo* in lung cancer cells. Tumors from the female nude mice were orthotopically transplanted with the same number of MDA-MB-231 and sicPLA2*α*/MDA-MB-231 cells (5 × 10^6^/mouse) into the fat pad (*n*=10/group), and after 5 weeks, all the mice were killed and then were evaluated for (**a**) the primary tumors' volume, weight and growth rates for two groups and (**b**) the number of lung metastasis for two groups. (**c**) Western blot of the expression of EMT-associated markers (epithelial marker E-cadherin, mesenchymal markers N-cadherin, vimentin and *α*-SMA) in primary tumors for two groups. (**d**) Western blot of EMT-associated markers expression (epithelial marker E-cadherin, mesenchymal markers N-cadherin, vimentin and *α*-SMA) in metastases for two groups. (**e**) Immunohistochemistry of EMT-associated markers expression for two groups. (**f**) Immunofluorescence of the expression of EMT-associated markers for two groups. Data are represented as the mean±S.D., *n*=10. **P<0.01 and ***P<0.001. Scale bar, 1.0 mm

To investigate the function of cPLA2*α* in tumorigenesis and metastasis *in vivo*, we orthotopically transplanted siSCR/MDA-MB-231 and sicPLA2*α*/MDA-MB-231 cells into the fat pad of the nude mice (5 × 10^6^/mouse). The tumor of siSCR/MDA-MB-231 cell-injection group could be felt at 1 week after inoculation. Then every two days, the tumor size was measured. However, it took ~2 weeks after inoculation for the tumors of sicPLA2*α*/MDA-MB-231 cell-injection group to be palpable. At the sixth week after inoculation, when the first mouse had become moribund, we killed all the mice in two groups and evaluated the tumor sizes and weights. The result showed that the suppression of cPLA2*α* markedly inhibited tumorigenesis by reducing the tumor weight, volume and growth rate *in vivo* (*P*<0.0001; Figure 6a). The number of lung metastases in the sicPLA2*α*/MDA-MB-231 cell-injection group was significantly lower than that in the siSCR/MDA-MB-231 cell-injection group (*P*<0.0001; Figure 6b).

Next, we evaluated the correlation between cPLA2*α* and EMT-associated markers in primary tumors and metastases through western blots analysis (Figures 6c and d), immunohistochemistry (Figure 6e) and immunofluorescence (Figure 6f) using the tumors of the two groups. The results were consistent with the previous *in vitro* experimental results. Mesenchymal markers, such as N-cadherin, vimentin and *α*-SMA, were downregulated in the tumors of the sicPLA2*α*/MDA-MB-231 cell-injection group, whereas the epithelial marker E-cadherin was upregulated.

**Figure 7:**
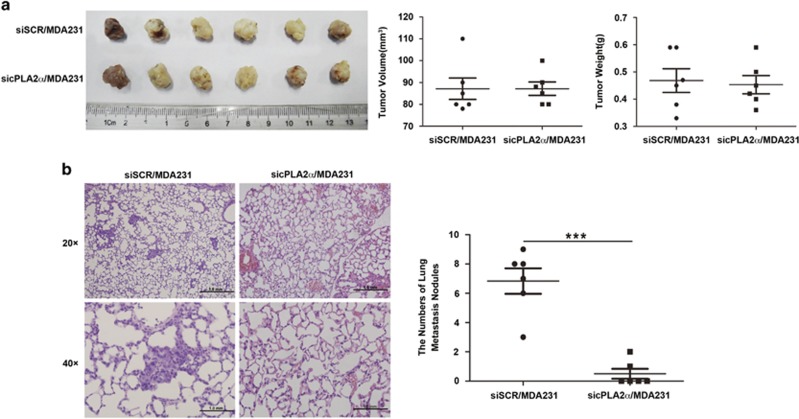
The reduction of breast cancer cell metastatic nodules in response to sicPLA2*α* was due to impaired metastatic seeding at the metastatic locus *in vivo*. Tumors from the female nude mice that were orthotopically transplanted with the same number of MDA-MB-231 and sicPLA2*α*/MDA-MB-231 cells (6 × 10^6^/mouse) into the fat pad (*n*=6/group), the mice were killed when the tumor volume reached ~1 cm^3^ (**a**) the primary tumor volume and weight for two groups and (**b**) the number of lung metastasis for two groups. Data are represented as the mean±S.D., *n*=6. ***P<0.001. Scale bar, 1.0 mm

Although, to further explore the phenomenon that the reduction in metastases in due to reduced tumor growth at primary site or due to impaired metastatic seeding at the metastatic locus, we designed an another group of animal experiment. We orthotopically transplanted siSCR/MDA-MB-231 and sicPLA2*α*/MDA-MB-231 cells into the fat pad of the nude mice (6 × 10^6^/mouse). The tumor volume was checked every two days with vernier calipers and the mice were killed when the tumor volume reached about 1 cm^3^. The result showed that when the two group mice tumor volume reached at the same volume and weight (Figure 7a), the reduction of metastatic nodules in response to sicPLA2*α* was still remarkable (*P*<0.0001; Figure 7b). This phenomenon could better explain that the reduction in metastases is due to impaired metastatic seeding at the metastatic locus.

Taken together, the results demonstrated that suppression of cPLA2*α* in MDA-MB-231 cells suppressed tumorigenesis and inhibited the lung metastasis of MDA-MB-231 cells *in vivo*.

## Discussion

In recent decades, with the development of gene sequencing technology for breast cancer, several targeted therapies interfering with specific molecules, according to different pathological classifications and genotyping, have been applied for clinical treatment.^39, 40^ However, metastasis is still the leading cause of death associated with breast cancer.^1^

It has been reported that cPLA2*α* is highly expressed in human prostate cancer,^41^ colon cancer,^24^ non-small-cell lung cancer and oral squamous carcinoma. In the current study, we found that cPLA2*α* was highly expressed in human breast cancerous tissues compared with the non-cancerous tissues and mammary gland floccules hyperplasia tissues. cPLA2*α* expression was closely associated with poor prognosis for HCC patients. Coincidentally, it has been shown that suppression of cPLA2*α* expression inhibits tumor cell proliferation in colon cancer.^26^ Ogawa and co-worders.^42^ demonstrated that the expression of membrane-associated phospholipase A2 is associated with breast cancer's malignant potency.^42^ However, the relationship between cPLA2*α* and breast cancer and the function of cPLA2*α* in regulating cancer cell metastasis have not been previously investigated.

Chronic inflammation has been implicated in a multitude of human malignancies.^5, 43, 44^ Among multifaceted links between chronic inflammation and cancer, COX2/PGE2 pathway has been the one to attract the most attention.^28, 45, 46^ cPLA2*α* catalyzes the hydrolysis of AA, which is enzymatically converted to PGE2 by the action of COX or LOX.^16^ Recently, direct evidence has shown that AA can promote human breast cancer cell migration and EMT through FAK and Src, respectively.^47, 48^ As the rate-limiting enzyme of AA production, cPLA2*α* has been demonstrated to have a fundamental role in gastric tumorigenesis as well as COX2.^49^ Nemenoff and co-workers^50^ demonstrated that despite an identical number of cells being injected, there was a dramatic decrease in the numbers of secondary metastatic tumors in cPLA2*α*-knockout mice.^50^ In this study, we showed that cPLA2*α* promoted human breast cancer cell migration and invasion by activating the EMT process.

EMT is an important event in tumor progression.^51^ Many studies have revealed that EMT is regulated by a group of transcription factors, such as Snail, Slug, Twist and ZEB1/2.^52, 53^ MET describes the reverse process of EMT.^54, 55^ During tumor progression, EMT mediates the initial transformation from benign to invasive carcinoma, and MET is critical for colonization in the later stages of metastasis. Recent studies have demonstrated that the transcription factors Ovol1 and Ovol2 act as drivers of MET and a brake on EMT.^56^ Our previous results demonstrated that the expression of Ovol2 was significantly lower in HCC tissues than in adjacent non-cancerous tissues and correlated with HCC prognosis.^57^ Our current results showed that in cPLA2*α*-downregulated cells, the expression of Slug and Twist (EMT-related transcription factors) decreased, whereas the expression of Ovol1 and Ovol2 increased (MET-related transcription factors). These findings suggest that cPLA2*α* mediates breast cancer cell EMT through the transcription factors Slug, Twist, Ovol1 and Ovol2.

The PI3K/Akt/GSK-3*β*/*β*-catenin signaling pathway can regulate the EMT process to influence tumor invasion and metastasis.^58^ Our study showed that inhibition of PI3K by LY294002 in cPLA2*α*-overexpressing MDA-MB-231 and T47D cells blocked the phosphorylation of Akt, GSK-3*β* and FAK. Moreover, we further found that overexpressed cPLA2*α* in MDA-MB-231 cells promoted *β*-catenin nuclear translocation and it was necessary for MDA-MB-231 cells' EMT translation. These results indicate that cPLA2*α* mediates TGF-*β*-induced EMT in human breast cancer cells via the PI3K/Akt/GSK-3*β*/*β*-catenin signaling pathway, ultimately improving their invasive and migratory potential.

In conclusion, we demonstrated that cPLA2*α* overexpression in breast cancer caused high metastasis and high malignancy features of the tumor and significantly affected the clinical outcomes of the patients. Our data showed that the suppression of cPLA2*α* expression in breast cancer cells significantly inhibited cancer cell migration and metastasis *in vitro* and *in vivo*. cPLA2*α* has a critical role in promoting cell invasion, metastasis and EMT via the PI3K/Akt pathway and can be expected to be a potential therapeutic target for breast cancer.

## Materials and Methods

### Clinical samples and cell culture

All patients provided written informed consent before we obtained the samples that were used in this study. The clinical samples mentioned in this research were all obtained from Tianjin Medical University Cancer Hospital. This study was conducted in accordance with Declaration of Helsinki and was approved by the Tianjin Medical University Cancer Hospital Ethics Committee. All breast cancer cell lines used in this study were purchased from the American Type Culture Collection (Manassas, VA, USA). MDA-MB-231, T47D and three stably transfected cell lines, sicPLA2*α*/MDA-MB-231, overcPLA2*α*/MDA-MB-231 and overcPLA2*α*/T47D, were all cultured in RPMI-1640 (Gibco, Carlsbad, CA, USA) supplemented with 10% fetal bovine serum (FBS; HyClone Laboratories Inc., Novato, CA, USA) and 1% penicillin–streptomycin solution (PS; HyClone). The MCF-10A cell line was cultured in DMEM/F-12 (Gibco) supplemented with horse serum (5% Invitrogen, Carlsbad, CA, USA), hydrocortisone (0.5 *μ*g/ml; Sigma, St. Louis, MO, USA), cholera toxin (0.1 *μ*g/ml; Sigma) and insulin (10 *μ*g/ml; Sigma). All cells were cultured in a humidified incubator maintained at 37 °C and 5% CO_2_.

### Western blot and antibodies

Western blot assays were performed as described previously.^59^ Briefly, the cells were serum-starved for 12 h. TGF-*β* (Peprotech, Rocky Hill, NJ, USA; 5 ng/ml, 48 h) and LY294002 (Sigma; 10 *μ*mol, 24 h) were added, and the cells were cultured at 37 °C in 5% CO_2_. The cells were washed three times with ice-cold phosphate-buffered saline (PBS) (pH 6.8) and then lysed with 1 × SDS lysis buffer (Tris-HCl, pH 6.8, 62.5 mM, 2% SDS, 10% glycerol) supplemented with 1 mM NaF, 1 mM Na_3_VO_4_, 1 × protease and phosphatase inhibitor cocktail (Hoffman-la Roche Ltd, Basel, Switzerland) on ice for 20 min. Protein denaturation was performed at 95 °C for 10 min, followed by centrifugation at 12 000 × *g* at 4 °C for 10 min, and then, the upper clear cell lysates were transferred. Equal amounts of protein (30–90 *μ*g/line) were loaded, separated by SDS-PAGE and transferred onto nitrocellulose membranes (Immobilon-P; Millipore, Billerica, MA, USA). Antibodies against the following proteins were used: E-cadherin (1:1000) from BD Biosciences (San Jose, CA, USA); GAPDH (1:1000), N-cadherin (1:500), ZEB1 (1:100), Ovol2 (1:200) and Slug (1:100) from Santa Cruz Biotechnology (Santa Cruz, CA, USA); vimentin (1:8000) from Epitomics (Burlingame, CA, USA); *α*-SMA (0.2 mg/ml) and Twist (1:1000) from Abcam (Hong Kong, China); Ovol1 (1:200) from Proteintech group (Chicago, IL, USA); cPLA2*α* (1:1000) from GeneTex (Irvine, TX, USA); Phospho-cPLA2*α* (1:1000), phospho-Akt (Ser473) (1:1000), phospho-FAK (1:1000), phospho-GSK-3*β* (1:1000) Akt (Ser473)(1:1000), FAK (1:1000) and GSK-3*β* (1:1000) from Cell Signaling Technology (Danvers, MA, USA); and goat anti-rabbit, goat anti-mouse and donkey anti-goat secondary antibody from Santa Cruz Biotechnology (1:4000).

### Nuclear and cytoplasmic fractionation

Nuclear and cytoplasmic fractionation was performed with the NE-PER Nuclear and Cytoplasmic Extraction Reagents (Thermo, Rockford, IL, USA). The steps were followed according to the user manual of NE-PER Nuclear and Cytoplasmic Extraction Reagents.

### Transfections

Transfections were performed with the Lenti-Pac HIV Expression Packaging Kit (GeneCopoeia, Rockville, MD, USA). The transfection steps were followed according to the user manual of Lenti-Pac HIV Expression Packaging Kit.

### RT-PCR

Total RNA from cells was extracted using TRIzol reagent (Invitrogen). Then, the One-Step RNA PCR Kit (AMV) from TaKaRa Biotechnology Co. Ltd (Dalian, China) was used to transcribe RNA. The amplification reaction needed 35 cycles. Each cycle contained denaturation at 95 °C for 1 min, annealing for 45 s and an extension at 72 °C for 1 min. A final extension step at 72 °C for 5 min terminated the amplification. Specific primers for GAPDH (forward, 5′-ACCACAGTCCATGCCATCAC-3′ reverse, 5′-TCCACCACCCTGTTGCTGTA-3′) and cPLA2*α* (forward, 5′-TCACATTTAACCTGCCGTATC-3′ reverse, 5′-CACTCCTTCAGCCCTTCC-3′) were also from TaKaRa Biotechnology Co. Ltd.

### Cell proliferation assay

The cells grew in 96-well plates at a density of 2 × 10^3^ cells per well for initial concentration. To reduce differences within the group, each group of cells sample a set of five parallel holes. Then, the cells were incubated with Cell Counting Kit-8 (Dojindo Laboratories, Kumamoto, Japan; 10 *μ*l/well) for 4 h in 37 °C and 5% CO_2_. The optical density was measured by an ELISA reader (BioTek SynergyH1, Burlington, VT, USA).

### Cell apoptosis assay by flow cytometry analysis

FITC Annexin V Apoptosis Detection Kit (BD Pharmingen, San Jose, CA, USA) was used to test the apoptosis rate of each group by flow cytometry analysis. The cells grew in 6 cm dish at a density of 1 × 10^6^ cells per well for appropriate concentration, and then washed the cells two times with cold PBS and resuspended cells in 1 × Binding Buffer. Transfered 100 *μ*l of the solution (1 × 10^5^ cells) to a 1.5 ml tube, added 5 *μ*l FITC Annexin V and 5 *μ*l PI. Gently vortexed the cells and incubated for 15 min at room temperature in the dark. Finally, added 400 *μ*l 1 × Binding Buffer to each tube and analyzed by flow cytometry in 1 h.

### Immunohistochemical assays

Xylene and graded concentrations of ethanol were used for sequential washing of the paraffin-embedded samples of invasive breast cancer. Endogenous peroxidase activity and nonspecific staining were blocked by 3% H_2_O_2_ for 15 min and 3% bovine serum albumin (BSA; Roche, Hong Kong, China) for 1 h, respectively. The incubation with the cPLA2*α* (N-216) (1:80) (Santa Cruz Biotechnology), E-cadherin (1:20) (BD Biosciences), N-cadherin (1:50) (Santa Cruz Biotechnology), or vimentin (1:1000) (Epitomics) antibodies occurred at room temperature for half an hour and then at 4 °C overnight. The tissues were washed with PBS three times and then stained with the secondary antibody (1:200) (Zhongshan, Beijing, China) at 37 °C for 1 h, visualized by 3,3′-diaminobenzidine staining and then counterstained with 10% Mayer hematoxylin, dehydrated, mounted, dried and observed.

The staining intensity of cPLA2*α* was classified on the scale of 0–3 (0 for no staining, 1 for weak immunoreactivity, 2 for medium immunoreactivity and 3 for strong immunoreactivity). Percentage immunoreactivity was scored on a scale from 0 to 3 (0 for no positive cells, 1 for <30% positive cells, 2 for 30 to 60% positive cells and 3 for >60% positive cells). The scores of staining intensity and percentage immunoreactivity were multiplied as the final scores of positive staining. We finally classified four expression levels of the staining: negative (score=0); weakly positive (+) (score=1–3); medium positive (++) (score=4–6); and strong positive (+++) (score=7–9). All images were captured with political fluorescence microscope (Olympus BX61, Tokyo, Japan).

### Immunofluorescence

Sterile coverslips were placed into 12-well plates, and then 4 × 10^4^ cells were plated in each well. After incubating at 37 °C in 5% CO_2_ for 12 h, the cells were fixed with 4% paraformaldehyde, permeabilized in 2% Triton X-100 and blocked in 3% BSA at room temperature for 1 h. Cells were incubated with E-cadherin (1:20) (BD Biosciences), N-cadherin (1:200) (Santa Cruz Biotechnology) and vimentin (1:5000) (Epitomics) antibodies overnight at 4 °C and washed two times with PBS. Cells were then stained with an Alexa Fluor 488-conjugated (Invitrogen) secondary antibody at room temperature for 1 h in a dark box. Cell nuclei were counterstained with prolong gold anti-fade reagent (Invitrogen). All images were captured with confocal laser scanning microscopy (Olympus FV1000).

### Frozen tissue immunofluorescence

The tissues were cryopreserved under the −80 °C and recovered at room temperature for 5 min. Tissues were then fixed in ice acetone at room temperature for 10 min, washed three times with PBS and blocked in 3% BSA at 37 °C for 1 h. The tissues were then incubated with E-cadherin (1:20) (BD Biosciences), N-cadherin (1:200) (Santa Cruz Biotechnology) and vimentin (1:5000) (Epitomics) antibodies overnight at 4 °C and washed three times with PBS immediately when transferred to room temperature. Then, the tissues were stained with Alexa Fluor 488- and 594-conjugated (Invitrogen) secondary antibodies at 37 °C for 1 h in a dark box and washed three times with PBS; the nuclei of the tissues were counterstained with prolong gold anti-fade reagent (Invitrogen). All images were captured with confocal laser scanning microscopy (Olympus FV1000).

### Chemotaxis assay

Briefly, RPMI-1640 containing 20% FBS was loaded into the lower chambers (30 *μ*l per chamber) and spread on the 8-*μ*m polyvinyl pyrrolidone-free polycarbonate filter membrane on the lower chambers, and then the cells were suspended in RPMI-1640 only at a density of 5 × 10^5^ cells/ml and loaded into the upper chambers (50 *μ*l per chamber). After incubating at 37 °C in 5% CO_2_ (siSCR/MDA-MB-231, sicPLA2*α*/MDA-MB-231, overSCR/MDA231 and overcPLA2*α*/MDA-MB-231 cells for 4 h; overSCR/T47D and overcPLA2*α*/T47D cells for 6 h), the membrane was rinsed with PBS, fixed in 4% paraformaldehyde and stained. Three fields were randomly selected to count the number of migrated cells at × 400 by light microscopy. All images were captured with a universal research motorized fluorescent microscopy (Olympus BX61).

### Matrigel invasion assay

Briefly, Matrigel (BD)-coated transwell chambers were prepared before the experiment. Then, 1 × 10^5^ cells in RPMI-1640 only were plated into the upper chamber with 8-*μ*m pores, and RPMI-1640 supplemented with EGF (Peprotech; 10 ng/ml) was added to the lower chamber. After incubation at 37 °C in 5% CO_2_ (siSCR/MDA-MB-231, sicPLA2*α*/MDA-MB-231, overSCR/MDA231 and overcPLA2*α*/MDA-MB-231 cells for 24 h; overSCR/T47D and overcPLA2*α*/T47D cells for 48 h), the invaded cells were fixed with 4% paraformaldehyde and stained. Three randomly selected fields were imaged, and the number of the cells was counted. All images were captured with a universal research motorized fluorescent microscopy (Olympus BX61).

### Scratch assay

Cells were plated in six-well plates one day before the assay. Each well was plated at a density of 1 × 10^6^ cells/ml to a fluent monolayer. The culture medium used was RPMI-1640 supplemented with 0.5% FBS. A 10-*μ*l pipette tip was used to generate an even wound. Then, the cells were cultured in a humidified incubator maintained at 37 °C and 5% CO_2_. The distance of the wounds was recorded at three random sites within an appropriate time (0, 3, 6, 9, 12 and 24 h). The data are shown as the mean±S.D. Images were captured at 0 and 24 h.

### Tumor xenograft transplantation assay

The mice were purchased from the Vital River Laboratory Animal Technology Co. Ltd (Beijing, China). All animal work procedures were approved by the Ethics Committee of the Tianjin Medical University Cancer Institute and Hospital, China. Four-week-old nude mice were used for xenograft animal experiments. Then, 5 × 10^6^ cells/ml of siSCR/MDA-MB-231 and sicPLA2*α*/MDA-MB-231 cells in 100 *μ*l of PBS were orthotopic transplanted into the mammary fat pads (*n*=10/group). The tumor volume was checked every two days with Vernier calipers. Both groups of mice were killed when the first mouse became moribund. The primary tumor weights and sizes were statistically analyzed. To examine the number of lung metastasis nodules, hematoxylin and eosin staining was used after the lungs were treated with formalin-fixed paraffin-embedded and serial sections.

## Figures and Tables

**Table 1:** Clinicopathological characteristics of patients with different cPLA2*α* expression levels undergoing surgery for breast cancer

**Clinicopathological characteristics**	***N* (%)**	**cPLA2***α* **expression**		***P*-value**
		**Low expression**	**High expression**	
*Age (years)*				
⩽50	50	29	21	0.785
>50	76	41	35	
				
*Tumor size (cm)*				
⩽2	43	34	9	<0.001
>2	83	36	47	
				
*Lymph node metastases*				
No	71	48	23	0.002
Yes	55	22	33	
				
*Relapse*				
No	109	65	44	0.020
Yes	17	5	12	
				
*ER expression*				
−	36	17	19	0.234
+	90	53	37	
				
*PR expression*				
−	58	32	26	0.936
+	68	38	30	
				
*CerbB2 expression*				
−	98	57	41	0.270
+	28	13	15	
				
*Ki67 expression*				
−	3	0	3	0.085
+	123	70	53	
				
*P53 expression*				
−	72	41	31	0.717
+	54	29	25	
				
*VEGF expression*				
−	51	31	20	0.330
+	75	39	36	
				
*Clinical grade*				
Stage 1	28	27	8	0.001
Stage 2	71	35	29	
Stage 3	27	8	19	
				
*Pathological grade*				
Luminal A	78	47	31	0.785
Luminal B	12	7	5	
HER2-enriched	16	8	8	
Triple negative	20	10	10	

Abbreviations: ER, estrogen receptor; HER2, human epidermal growth factor receptor-2; cPLA2*α*, Cytosolic phospholipase A2*α*; PR, progesterone receptor; VEGF, vascular endothelial growth factor

## References

[bib1] Siegel R, Ma J, Zou Z, Jemal A. Cancer statistics. Cancer J Clin 2014; 64: 9–29. 10.3322/caac.21208 24399786

[bib2] Ma H, Ursin G, Xu X, Lee E, Togawa K, Duan L et al. Reproductive factors and the risk of triple-negative breast cancer in white women and African-American women: a pooled analysis. Breast Cancer Res 2017; 19: 6. 28086982 10.1186/s13058-016-0799-9 PMC5237290

[bib3] Park S, Han W, Kim J, Kim MK, Lee E, Yoo TK et al. Risk factors associated with distant metastasis and survival outcomes in breast cancer patients with locoregional recurrence. J Breast Cancer 2015; 18: 160–166. 26155292 10.4048/jbc.2015.18.2.160 PMC4490265

[bib4] Vera-Ramirez L, Sanchez-Rovira P, Ramirez-Tortosa CL, Quiles JL, Ramirez-Tortosa MC, Alvarez JC et al. Gene-expression profiles, tumor microenvironment, and cancer stem cells in breast cancer: latest advances towards an integrated approach. Cancer Treat Rev 2010; 36: 477–484. 20299155 10.1016/j.ctrv.2010.02.017

[bib5] Coussens LM, Werb Z. Inflammation and cancer. Nature 2002; 420: 860–867. 12490959 10.1038/nature01322 PMC2803035

[bib6] Chua HL, Bhat-Nakshatri P, Clare SE, Morimiya A, Badve S, Nakshatri H. NF-kappaB represses E-cadherin expression and enhances epithelial to mesenchymal transition of mammary epithelial cells: potential involvement of ZEB-1 and ZEB-2. Oncogene 2007; 26: 711–724. 16862183 10.1038/sj.onc.1209808

[bib7] Kim HJ, Litzenburger BC, Cui X, Delgado DA, Grabiner BC, Lin X et al. Constitutively active type I insulin-like growth factor receptor causes transformation and xenograft growth of immortalized mammary epithelial cells and is accompanied by an epithelial-to-mesenchymal transition mediated by NF-kappaB and snail. Mol Cell Biol 2007; 27: 3165–3175. 17296734 10.1128/MCB.01315-06 PMC1899918

[bib8] Mantovani A, Allavena P, Sica A, Balkwill F. Cancer-related inflammation. Nature 2008; 454: 436–444. 18650914 10.1038/nature07205

[bib9] Landskron G, De la Fuente M, Thuwajit P, Thuwajit C, Hermoso MA. Chronic inflammation and cytokines in the tumor microenvironment. J Immunol Res 2014; 2014: 149185. 24901008 10.1155/2014/149185 PMC4036716

[bib10] Uozumi N, Kume K, Nagase T, Nakatani N, Ishii S, Tashiro F et al. Role of cytosolic phospholipase A2 in allergic response and parturition. Nature 1997; 390: 618–622. 9403692 10.1038/37622

[bib11] Villegas-Comonfort S, Serna-Marquez N, Galindo-Hernandez O, Navarro-Tito N, Salazar EP. Arachidonic acid induces an increase of beta-1,4-galactosyltransferase I expression in MDA-MB-231 breast cancer cells. J Cell Biochem 2012; 113: 3330–3341. 22644815 10.1002/jcb.24209

[bib12] Park SY, Jeong KJ, Panupinthu N, Yu S, Lee J, Han JW et al. Lysophosphatidic acid augments human hepatocellular carcinoma cell invasion through LPA1 receptor and MMP-9 expression. Oncogene 2011; 30: 1351–1359. 21102517 10.1038/onc.2010.517

[bib13] Zhang Z, Lee YC, Kim SJ, Choi MS, Tsai PC, Saha A et al. Production of lysophosphatidylcholine by cPLA2 in the brain of mice lacking PPT1 is a signal for phagocyte infiltration. Hum Mol Genet 2007; 16: 837–847. 17341491 10.1093/hmg/ddm029

[bib14] Falasca M, Ferro R. Role of the lysophosphatidylinositol/GPR55 axis in cancer. Adv Biol Regul 2016; 60: 88–93. 26588872 10.1016/j.jbior.2015.10.003

[bib15] Pandey R, Ghorpade A. HIV-1 and alcohol abuse promote astrocyte inflammation: a mechanistic synergy via the cytosolic phospholipase A2 pathway. Cell Death Dis 2015; 6: e2017. 10.1038/cddis.2015.346 PMC4720882 26658191

[bib16] Leslie CC. Cytosolic phospholipase A2: physiological function and role in disease. J Lipid Res 2015; 56: 1386–1402. 25838312 10.1194/jlr.R057588 PMC4513982

[bib17] Czachorowski MJ, Amaral AF, Montes-Moreno S, Lloreta J, Carrato A, Tardon A et al. Cyclooxygenase-2 expression in bladder cancer and patient prognosis: results from a large clinical cohort and meta-analysis. PLoS ONE 2012; 7: e45025. 23028744 10.1371/journal.pone.0045025 PMC3441520

[bib18] Ogunwobi OO, Liu C. Hepatocyte growth factor upregulation promotes carcinogenesis and epithelial–mesenchymal transition in hepatocellular carcinoma via Akt and COX-2 pathways. Clin Exp Metastasis 2011; 28: 721–731. 21744257 10.1007/s10585-011-9404-x PMC3732749

[bib19] Jang TJ. Epithelial to mesenchymal transition in cutaneous squamous cell carcinoma is correlated with COX-2 expression but not with the presence of stromal macrophages or CD10-expressing cells. Virchows Archiv 2012; 460: 481–487. 22460857 10.1007/s00428-012-1227-x

[bib20] Bocca C, Bozzo F, Miglietta A. COX2 inhibitor NS398 reduces HT-29 cell invasiveness by modulating signaling pathways mediated by EGFR and HIF1-alpha. Anticancer Res 2014; 34: 1793–1800. 24692712

[bib21] Vignarajan S, Xie C, Yao M, Sun Y, Simanainen U, Sved P et al. Loss of PTEN stabilizes the lipid modifying enzyme cytosolic phospholipase A(2)alpha via AKT in prostate cancer cells. Oncotarget 2014; 5: 6289–6299. 25026288 10.18632/oncotarget.2198 PMC4171630

[bib22] Herbert SP, Odell AF, Ponnambalam S, Walker JH. Activation of cytosolic phospholipase A2-{alpha} as a novel mechanism regulating endothelial cell cycle progression and angiogenesis. J Biol Chem 2009; 284: 5784–5796. 19119141 10.1074/jbc.M807282200 PMC2645829

[bib23] Schulte RR, Linkous AG, Hallahan DE, Yazlovitskaya EM. Cytosolic phospholipase A2 as a molecular target for the radiosensitization of ovarian cancer. Cancer Lett 2011; 304: 137–143. 21397389 10.1016/j.canlet.2011.02.015 PMC3075208

[bib24] Zheng Z, He X, Xie C, Hua S, Li J, Wang T et al. Targeting cytosolic phospholipase A2 alpha in colorectal cancer cells inhibits constitutively activated protein kinase B (AKT) and cell proliferation. Oncotarget 2014; 5: 12304–12316. 25365190 10.18632/oncotarget.2639 PMC4322978

[bib25] Yu Y, Zhang X, Hong S, Zhang M, Cai Q, Jiang W et al. Epidermal growth factor induces platelet-activating factor production through receptors transactivation and cytosolic phospholipase A2 in ovarian cancer cells. J Ovarian Res 2014; 7: 39. 24721622 10.1186/1757-2215-7-39 PMC4005630

[bib26] Kisslov L, Hadad N, Rosengraten M, Levy R. HT-29 human colon cancer cell proliferation is regulated by cytosolic phospholipase A(2)alpha dependent PGE(2)via both PKA and PKB pathways. Biochim Biophys Acta 2012; 1821: 1224–1234. 22728329 10.1016/j.bbalip.2012.06.005

[bib27] Gentile LB, Piva B, Capizzani BC, Furlaneto LG, Moreira LS, Zamith-Miranda D et al. Hypertonic environment elicits cyclooxygenase-2-driven prostaglandin E2 generation by colon cancer cells: role of cytosolic phospholipase A2-alpha and kinase signaling pathways. Prostaglandins Leukot Essent Fatty Acids 2010; 82: 131–139. 20004562 10.1016/j.plefa.2009.11.005

[bib28] Grewal S, Ponnambalam S, Walker JH. Association of cPLA2-alpha and COX-1 with the Golgi apparatus of A549 human lung epithelial cells. J Cell Sci 2003; 116(Part 11): 2303–2310. 12711701 10.1242/jcs.00446

[bib29] Baek SH, Ko JH, Lee JH, Kim C, Lee H, Nam D et al. Ginkgolic acid inhibits invasion and migration and TGF-beta-induced EMT of lung cancer cells through PI3K/Akt/mTOR inactivation. J Cell Physiol 2017; 232: 346–354. 27177359 10.1002/jcp.25426

[bib30] Zhu C, Sun Z, Li C, Guo R, Li L, Jin L et al. Urocortin affects migration of hepatic cancer cell lines via differential regulation of cPLA2 and iPLA2. Cell Signal 2014; 26: 1125–1134. 24518041 10.1016/j.cellsig.2014.02.002

[bib31] Reichl P, Haider C, Grubinger M, Mikulits W. TGF-beta in epithelial to mesenchymal transition and metastasis of liver carcinoma. Curr Pharm Des 2012; 18: 4135–4147. 22630087 10.2174/138161212802430477

[bib32] Xu J, Lamouille S, Derynck R. TGF-beta-induced epithelial to mesenchymal transition. Cell Res 2009; 19: 156–172. 19153598 10.1038/cr.2009.5 PMC4720263

[bib33] Zhao H, Lv F, Liang G, Huang X, Wu G, Zhang W et al. FGF19 promotes epithelial–mesenchymal transition in hepatocellular carcinoma cells by modulating the GSK3beta/beta-catenin signaling cascade via FGFR4 activation. Oncotarget 2016; 7: 13575–13586. 26498355 10.18632/oncotarget.6185 PMC4924662

[bib34] Liu L, Dai Y, Chen J, Zeng T, Li Y, Chen L et al. Maelstrom promotes hepatocellular carcinoma metastasis by inducing epithelial–mesenchymal transition by way of Akt/GSK-3beta/Snail signaling. Hepatology 2014; 59: 531–543. 23929794 10.1002/hep.26677

[bib35] Hsieh YS, Chu SC, Hsu LS, Chen KS, Lai MT, Yeh CH et al. *Rubus idaeus* L. reverses epithelial-to-mesenchymal transition and suppresses cell invasion and protease activities by targeting ERK1/2 and FAK pathways in human lung cancer cells. Food Chem Toxicol 2013; 62: 908–918. 24161487 10.1016/j.fct.2013.10.021

[bib36] Okada T, Sinha S, Esposito I, Schiavon G, Lopez-Lago MA, Su W et al. The Rho GTPase Rnd1 suppresses mammary tumorigenesis and EMT by restraining Ras-MAPK signalling. Nat Cell Biol 2015; 17: 81–94. 25531777 10.1038/ncb3082 PMC4374353

[bib37] Bravo-Cordero JJ, Magalhaes MA, Eddy RJ, Hodgson L, Condeelis J. Functions of cofilin in cell locomotion and invasion. Nat Rev Mol Cell Biol 2013; 14: 405–415. 23778968 10.1038/nrm3609 PMC3878614

[bib38] Zhang PF, Li KS, Shen YH, Gao PT, Dong ZR, Cai JB et al. Galectin-1 induces hepatocellular carcinoma EMT and sorafenib resistance by activating FAK/PI3K/AKT signaling. Cell Death Dis 2016; 7: e2201. 27100895 10.1038/cddis.2015.324 PMC4855644

[bib39] Toss A, Cristofanilli M. Molecular characterization and targeted therapeutic approaches in breast cancer. Breast Cancer Res 2015; 17: 60. 25902832 10.1186/s13058-015-0560-9 PMC4407294

[bib40] Wheler JJ, Atkins JT, Janku F, Moulder SL, Yelensky R, Stephens PJ et al. Multiple gene aberrations and breast cancer: lessons from super-responders. BMC Cancer 2015; 15: 442. 26021831 10.1186/s12885-015-1439-y PMC4446801

[bib41] Patel MI, Singh J, Niknami M, Kurek C, Yao M, Lu S et al. Cytosolic phospholipase A2-alpha: a potential therapeutic target for prostate cancer. Clin Cancer Res 2008; 14: 8070–8079. 19088022 10.1158/1078-0432.CCR-08-0566 PMC2605658

[bib42] Yamashita S, Yamashita J, Ogawa M. Overexpression of group II phospholipase A2 in human breast cancer tissues is closely associated with their malignant potency. Br J Cancer 1994; 69: 1166–1170. 8198986 10.1038/bjc.1994.229 PMC1969450

[bib43] Alison MR, Nicholson LJ, Lin WR. Chronic inflammation and hepatocellular carcinoma. Recent Results Cancer Res 2011; 185: 135–148. 21822824 10.1007/978-3-642-03503-6_8

[bib44] Crusz SM, Balkwill FR. Inflammation and cancer: advances and new agents. Nat Rev Clin Oncol 2015; 12: 584–596. 26122183 10.1038/nrclinonc.2015.105

[bib45] Montrose DC, Nakanishi M, Murphy RC, Zarini S, McAleer JP, Vella AT et al. The role of PGE2 in intestinal inflammation and tumorigenesis. Prostaglandins Other Lipid Mediat 2015; 116-117: 26–36. 25460828 10.1016/j.prostaglandins.2014.10.002 PMC4385488

[bib46] Larsson K, Kock A, Idborg H, Arsenian Henriksson M, Martinsson T, Johnsen JI et al. COX/mPGES-1/PGE2 pathway depicts an inflammatory-dependent high-risk neuroblastoma subset. Proc Natl Acad Sci USA 2015; 112: 8070–8075. 26080408 10.1073/pnas.1424355112 PMC4491767

[bib47] Navarro-Tito N, Robledo T, Salazar EP. Arachidonic acid promotes FAK activation and migration in MDA-MB-231 breast cancer cells. Exp Cell Res 2008; 314: 3340–3355. 18804105 10.1016/j.yexcr.2008.08.018

[bib48] Martinez-Orozco R, Navarro-Tito N, Soto-Guzman A, Castro-Sanchez L, Perez Salazar E. Arachidonic acid promotes epithelial-to-mesenchymal-like transition in mammary epithelial cells MCF10A. Eur J Cell Biol 2010; 89: 476–488. 20207443 10.1016/j.ejcb.2009.12.005

[bib49] Zhang X, Wu Q, Gan L, Yu GZ, Wang R, Wang ZS et al. Reduced group IVA phospholipase A2 expression is associated with unfavorable outcome for patients with gastric cancer. Med Oncol 2013; 30: 454. 23307260 10.1007/s12032-012-0454-y

[bib50] Weiser-Evans MC, Wang XQ, Amin J, Van Putten V, Choudhary R, Winn RA et al. Depletion of cytosolic phospholipase A2 in bone marrow-derived macrophages protects against lung cancer progression and metastasis. Cancer Res 2009; 69: 1733–1738. 19208832 10.1158/0008-5472.CAN-08-3766 PMC2653105

[bib51] Thiery JP, Acloque H, Huang RY, Nieto MA. Epithelial–mesenchymal transitions in development and disease. Cell 2009; 139: 871–890. 19945376 10.1016/j.cell.2009.11.007

[bib52] Lee TI, Young RA. Transcriptional regulation and its misregulation in disease. Cell 2013; 152: 1237–1251. 23498934 10.1016/j.cell.2013.02.014 PMC3640494

[bib53] Zheng X, Carstens JL, Kim J, Scheible M, Kaye J, Sugimoto H et al. Epithelial-to-mesenchymal transition is dispensable for metastasis but induces chemoresistance in pancreatic cancer. Nature 2015; 527: 525–53041456. 26560028 10.1038/nature16064 PMC4849281

[bib54] Esteban MA, Bao X, Zhuang Q, Zhou T, Qin B, Pei D. The mesenchymal-to-epithelial transition in somatic cell reprogramming. Curr Opin Genet Dev 2012; 22: 423–428. 23084025 10.1016/j.gde.2012.09.004

[bib55] Yang J, Du X, Wang G, Sun Y, Chen K, Zhu X et al. Mesenchymal to epithelial transition in sarcomas. Eur J Cancer 2014; 50: 593–601. 24291235 10.1016/j.ejca.2013.11.006

[bib56] Roca H, Hernandez J, Weidner S, McEachin RC, Fuller D, Sud S et al. Transcription factors OVOL1 and OVOL2 induce the mesenchymal to epithelial transition in human cancer. PLoS ONE 2013; 8: e76773. 24124593 10.1371/journal.pone.0076773 PMC3790720

[bib57] Fu H, Qi L, Chen L, He Y, Zhang N, Guo H. Expression of Ovol2 is related to epithelial characteristics and shows a favorable clinical outcome in hepatocellular carcinoma. OncoTargets Ther 2016; 9: 5963–5973. 10.2147/OTT.S110409 PMC5047729 27729805

[bib58] Grille SJ, Bellacosa A, Upson J, Klein-Szanto AJ, van Roy F, Lee-Kwon W et al. The protein kinase Akt induces epithelial mesenchymal transition and promotes enhanced motility and invasiveness of squamous cell carcinoma lines. Cancer Res 2003; 63: 2172–2178. 12727836

[bib59] Guo H, Gu F, Li W, Zhang B, Niu R, Fu L et al. Reduction of protein kinase C zeta inhibits migration and invasion of human glioblastoma cells. J Neurochem 2009; 109: 203–213. 19187446 10.1111/j.1471-4159.2009.05946.x

