# Supplementary material for: cPLA2α mediates TGF-β-induced epithelial–mesenchymal transition in breast cancer through PI3k/Akt signaling
Source: Cell Death Dis. 2017 Apr 6;8(4):e2728–. doi: 10.1038/cddis.2017.152 (PMC5477578; doi:10.1038/cddis.2017.152)

**Supplementary Figure 1 Either endogenous or exogenous cPLA2α inhibition induced breast cancer cells losing aggressive phenotypes.** (A) Chemotaxis and (B) Wound-healing assay of MDA-MB-231 cells cultured with or without cPLA2α and PLC inhibitor. (C) Chemotaxis of T47D cells cultured with or without cPLA2α inhibitor. (D) Western blot and qRT-PCR of MDA-MB-231 cells transfected with control siRNA (SCR) and cPLA2α siRNA (sicPLA2α＃1-3). Chemotaxis of (E) MDA-MB-231 cells (F) T47D cells transfected with or without SCR and sicPLA2α. ***p*<0.01, ****P*<0.001. All experiments were repeated at least three times.


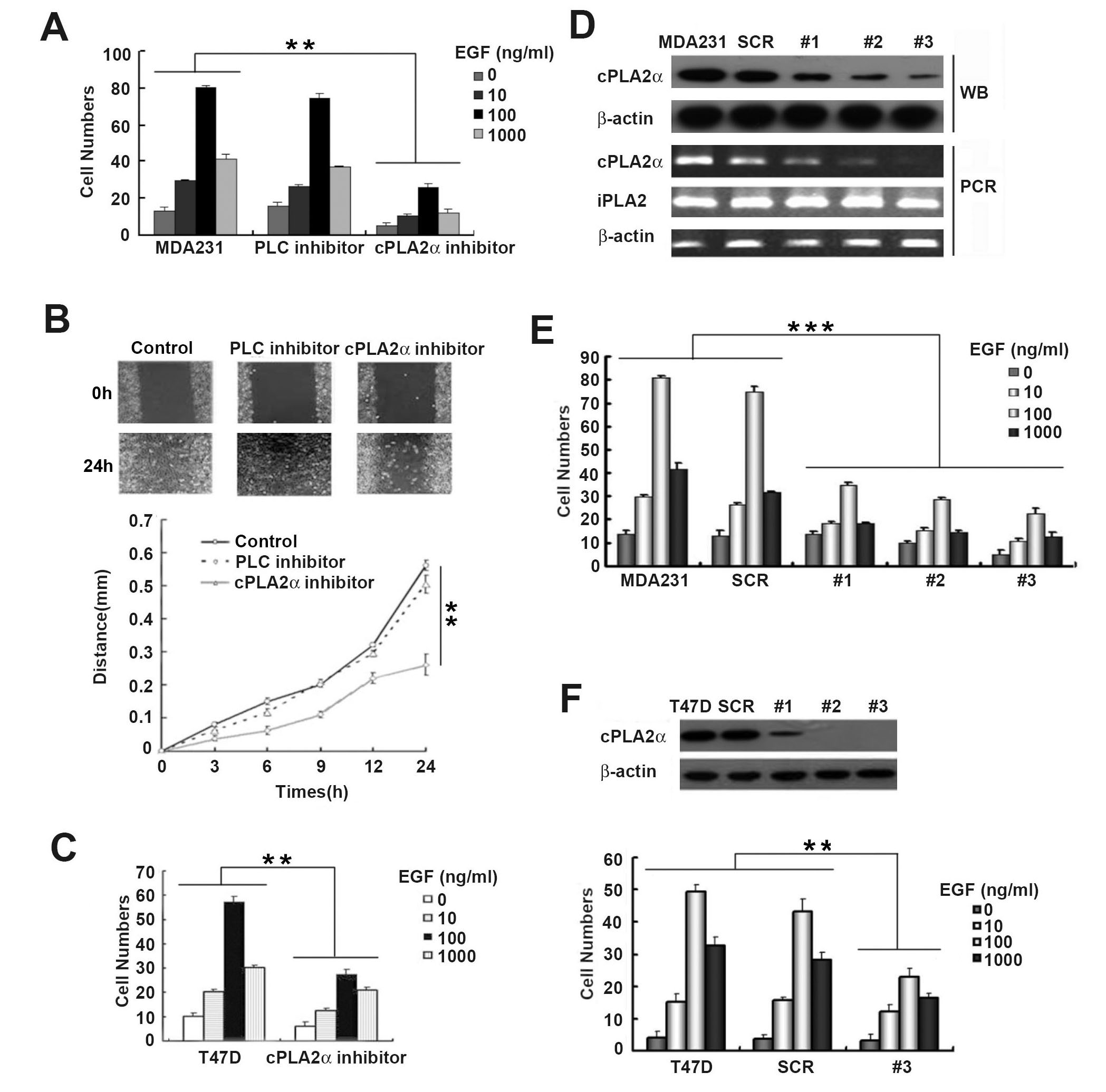

Supplement: Supplementary Figure 1 [file cddis2017152x1.docx]
