# Supplementary material for: cPLA2α mediates TGF-β-induced epithelial–mesenchymal transition in breast cancer through PI3k/Akt signaling
Source: Cell Death Dis. 2017 Apr 6;8(4):e2728–. doi: 10.1038/cddis.2017.152 (PMC5477578; doi:10.1038/cddis.2017.152)

**Supplementary Figure 2 Different expression levels of cPLA2α in MDA-MB-231 cells had no effect on cells survival.** Comparison of the apoptosis rates of (A) siSCR/MDA-MB-231 and sicPLA2α/MDA-MB-231 (B) overSCR/MDA-MB-231 and overcPLA2αMDA-MB-231 cells by flow cytometry analysis of Annexin V-FITC/PI.


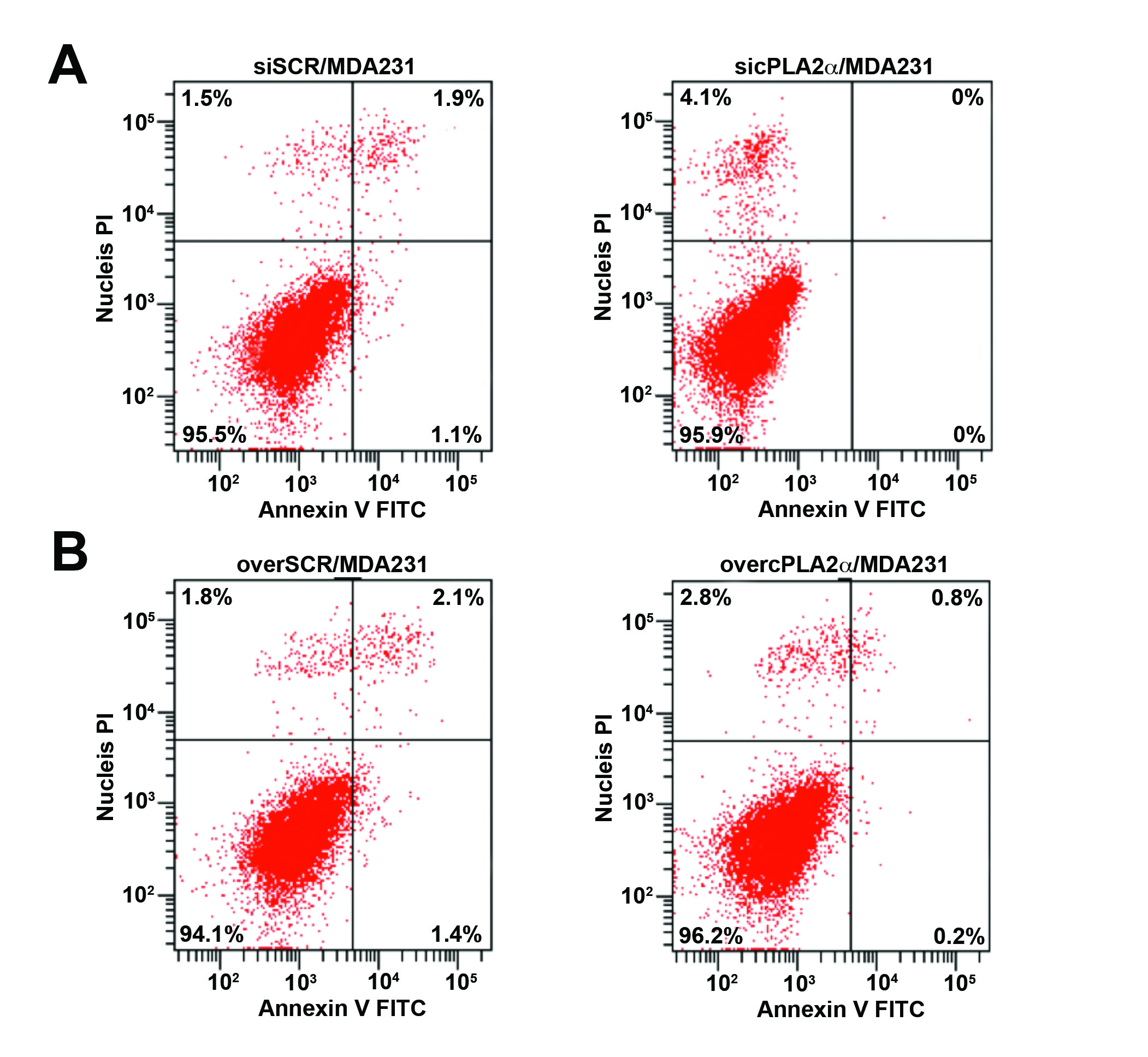

Supplement: Supplementary Figure 2 [file cddis2017152x2.docx]
