# Supplementary material for: cPLA2α mediates TGF-β-induced epithelial–mesenchymal transition in breast cancer through PI3k/Akt signaling
Source: Cell Death Dis. 2017 Apr 6;8(4):e2728–. doi: 10.1038/cddis.2017.152 (PMC5477578; doi:10.1038/cddis.2017.152)

**Supplementary Figure 3 cPLA2α induced breast cancer cells EMT process though β-Catenin nuclear translocation.** Western blot of β-Catenin in (A) siSCR/MDA-MB-231 and sicPLA2α/MDA-MB-231 cells when cultured without or with TGF-β (15ng/ml, 2h); (B) overSCR/MDA-MB-231 and overcPLA2α/MDA-MB-231 cells when cultured without or with TGF-β(15ng/ml, 2h); (C) Assessment of the transiently transfection efficiency of β-catenin protein expression in overcPLA2α/MDA231 cells; (D) Comparison of Chemotaxis potential of overcPLA2α/MDAMB-231 and overcPLA2αsiβ-catenin/MDAMB-231 cells, ***P<0.001; (E) Comparison of proliferation potential of overcPLA2α/MDAMB-231 and overcPLA2αsiβ-catenin/MDAMB-231 cells. All experiments were repeated at least three times.


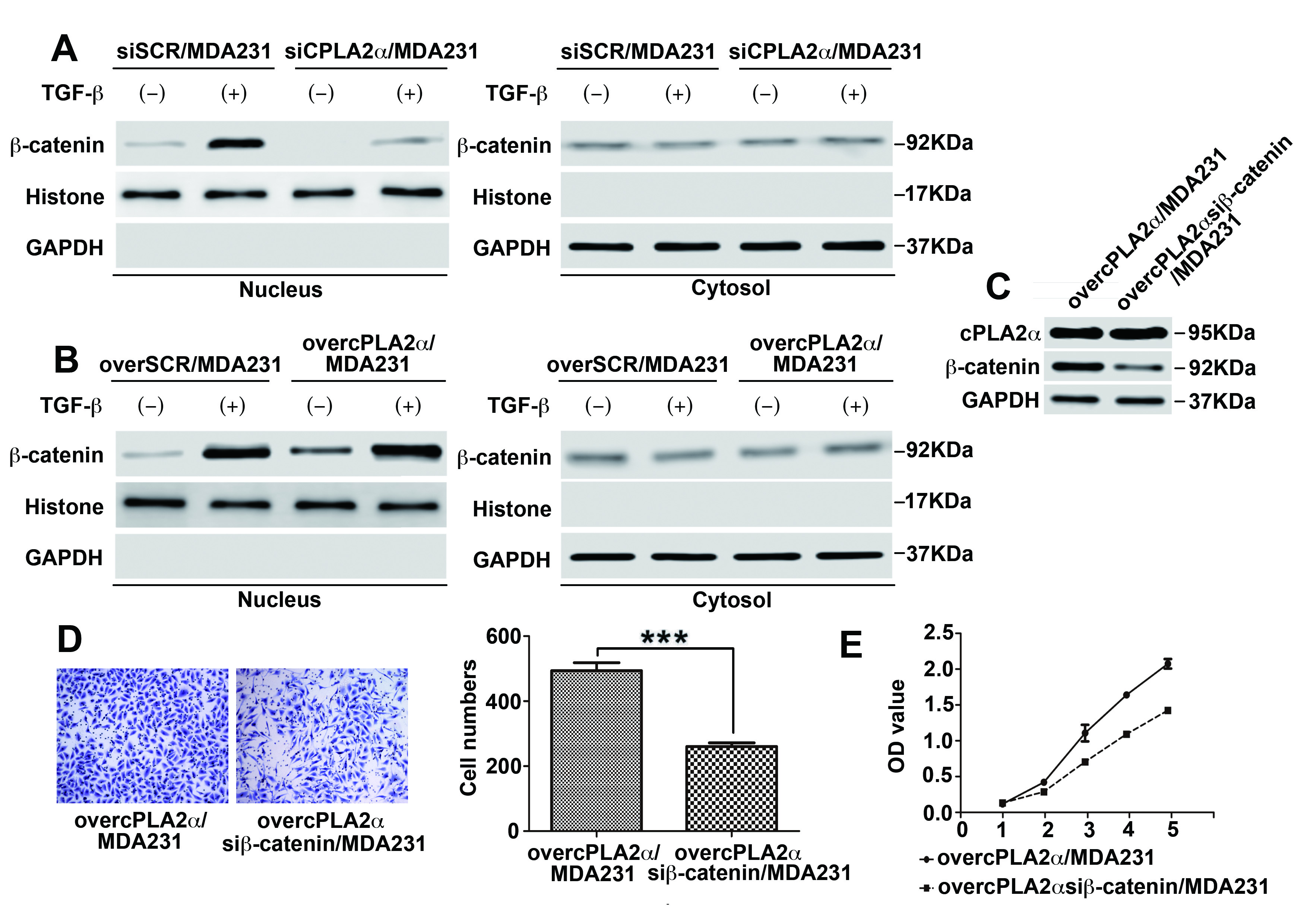

Supplement: Supplementary Figure 3 [file cddis2017152x3.docx]
